# Comparative transcriptomic analysis of the mechanisms underpinning ageing and fecundity in social insects

**DOI:** 10.1098/rstb.2019.0728

**Published:** 2021-04-26

**Authors:** Judith Korb, Karen Meusemann, Denise Aumer, Abel Bernadou, Daniel Elsner, Barbara Feldmeyer, Susanne Foitzik, Jürgen Heinze, Romain Libbrecht, Silu Lin, Megha Majoe, José Manuel Monroy Kuhn, Volker Nehring, Matteo A. Negroni, Robert J. Paxton, Alice C. Séguret, Marah Stoldt, Thomas Flatt, the So-Long consortium

**Affiliations:** ^1^ Department of Evolutionary Biology and Ecology, Institute of Biology I (Zoology), University of Freiburg, Hauptstraße 1, D-79104 Freiburg (Breisgau), Germany; ^2^ Australian National Insect Collection, CSIRO National Research Collections Australia, Clunies Ross Street, Canberra, Acton 2601, Australia; ^3^ Developmental Zoology, Molecular Ecology Research Group, Hoher Weg 4, D-06099 Halle (Saale), Germany; ^4^ Zoology/Evolutionary Biology, University of Regensburg, Universitätsstraße 31, D-93040 Regensburg, Germany; ^5^ Senckenberg Biodiversity and Climate Research Centre (SBiK-F), Molecular Ecology, Senckenberg, Georg-Voigt-Straße 14-16, D-60325 Frankfurt, Germany; ^6^ Institute of Organismic and Molecular Evolution (IOME), Johannes Gutenberg University Mainz, Hanns-Dieter-Hüsch-Weg 15, D-55128 Mainz, Germany; ^7^ Institute for Biology, Martin Luther University Halle-Wittenberg, Hoher Weg 8, 06120 Halle, Germany; ^8^ Institute for Evolution and Biodiversity, University of Münster, Hüfferstraße 1, 48149 Münster, Germany; ^9^ Department of Biology, University of Fribourg, Chemin du Musée 10, CH-1700 Fribourg, Switzerland

**Keywords:** social insects, longevity, insulin, TOR, juvenile hormone, transcriptomics

## Abstract

The exceptional longevity of social insect queens despite their lifelong high fecundity remains poorly understood in ageing biology. To gain insights into the mechanisms that might underlie ageing in social insects, we compared gene expression patterns between young and old castes (both queens and workers) across different lineages of social insects (two termite, two bee and two ant species). After global analyses, we paid particular attention to genes of the insulin/insulin-like growth factor 1 signalling (IIS)/target of rapamycin (TOR)/juvenile hormone (JH) network, which is well known to regulate lifespan and the trade-off between reproduction and somatic maintenance in solitary insects. Our results reveal a major role of the downstream components and target genes of this network (e.g. JH signalling, vitellogenins, major royal jelly proteins and immune genes) in affecting ageing and the caste-specific physiology of social insects, but an apparently lesser role of the upstream IIS/TOR signalling components. Together with a growing appreciation of the importance of such downstream targets, this leads us to propose the TI–J–LiFe (**T**OR/**I**IS–**J**H–**Li**fespan and **Fe**cundity) network as a conceptual framework for understanding the mechanisms of ageing and fecundity in social insects and beyond.

This article is part of the theme issue ‘Ageing and sociality: why, when and how does sociality change ageing patterns?’

## 1. Introduction

Why do organisms age? This is a major question in evolutionary biology, given that an unlimited lifespan associated with continuous reproduction would increase fitness and hence should be favoured. The classical evolutionary theory of ageing, developed by Medawar, Williams and Hamilton [1–3], has, in principle, explained why ageing evolves. However, we still understand very little about the tremendous diversity of ageing rates among organisms and the mechanisms that might underlie this diversity [4] (reviewed in [5,6]).

**Figure 1.**
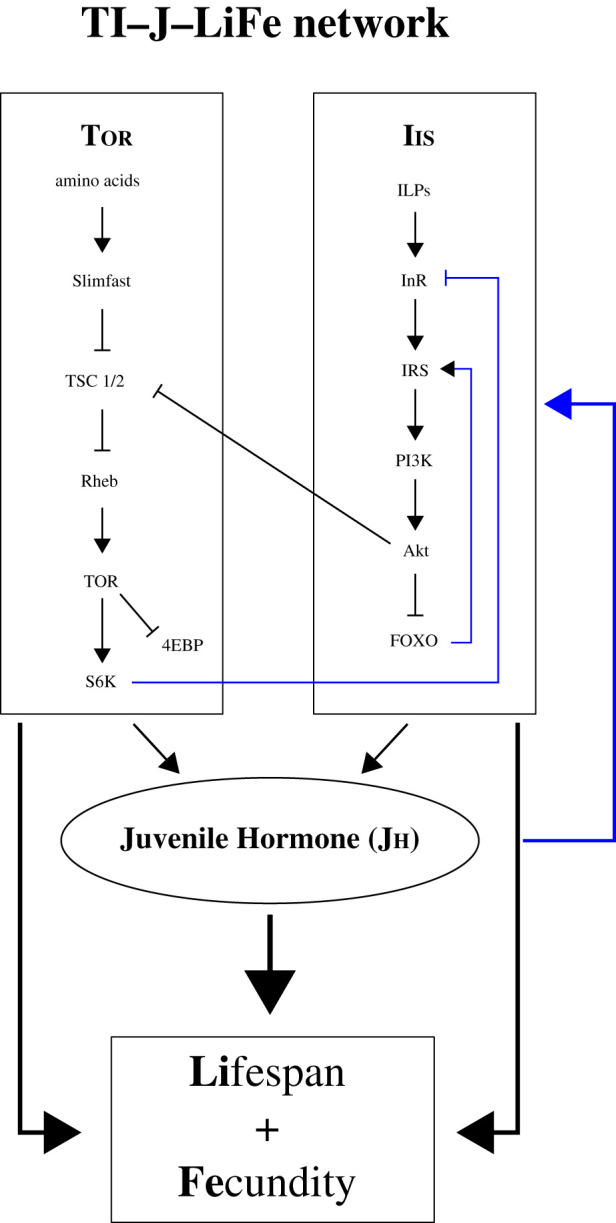
The ‘TI–J–LiFe’ network. The **TI–J–LiFe** network represents a set of interacting pathways that comprise the nutrient sensing **T**OR (target of rapamycin) and **I**IS (insulin/insulin-like growth factor 1 signalling) pathways, the **J**uvenile Hormone (JH, a major lipophilic hormone whose production is regulated by IIS and TOR), as well as downstream processes targeted by this network, including somatic maintenance functions (e.g. immunity and oxidative stress resistance) and reproductive physiology (including vitellogenins and yolk proteins), that have profound effects upon insect life history, especially on **Li**fespan and **Fe**cundity. This network is thought to be one of the major regulatory circuits underpinning variation of insect lifespan and the trade-off between fecundity and longevity. The core components and feedback loops depicted here are mainly based on experimental findings in *Drosophila melanogaster* (for detailed information, see https://flybase.org; e.g. IIS gene lists at: https://flybase.org/reports/FBgg0000904.html; https://flybase.org/reports/FBgg0000900.html; https://flybase.org/reports/FBgg0000898.html). Previous work suggests that this network and its effects are evolutionarily highly conserved among insects beyond *Drosophila*. In some social insects (e.g. *Apis mellifera*), some parts of this network might be ‘wired’ differently, but whether such a ‘rewiring’ is common among social insects remains largely unknown (for further discussion, see [18]). (Online version in colour.)

During the last decades, results from model organisms have revealed the existence of a conserved set of gene networks and pathways involved in ageing in animals ranging from nematodes and flies to mice and humans (see [6–20], and references therein). In many insects, for example, the insulin/insulin-like growth factor 1 signalling (IIS)/target of rapamycin (TOR)/juvenile hormone (JH) network has emerged as a key regulator of lifespan and somatic maintenance, growth and fecundity, and explains trade-offs between these processes (figure 1). The IIS and TOR pathways sense the availability of nutrients, such as carbohydrates and amino acids. Through a cascade of signalling activities, they positively affect the production of the lipophilic sesquiterpenoid hormone JH (as well as the steroid hormone 20-hydroxy-ecdysone) and regulate various physiological processes including reproductive physiology (e.g. egg maturation, by affecting the expression of yolk proteins or the yolk precursor protein vitellogenin; see [13–19]), somatic maintenance (e.g. humoral innate immunity and oxidative stress resistance) and lifespan (see reviews in [6–20] and references therein). In particular, results from the fruit fly *Drosophila melanogaster* as well as from other relatively short-lived insects (e.g. grasshoppers, butterflies, bugs and planthoppers) suggest that downregulation of this signalling network (e.g. via experimental ablation of insulin-producing cells or of the gland that produces JH) promotes somatic maintenance and longevity at the expense of fecundity (e.g. [7,13,15–17,20] and references therein). Because of its central role in modulating insect life history and ageing, we herein refer to this integrated network and the downstream processes that it affects as the TI–J–LiFe network (**T**OR/**I**IS–**J**H–**Li**fespan and **Fe**cundity) (figure 1).

Considerably less is known, however, about the role of this signalling system in affecting ageing of social insects in which queens have extraordinarily long lifespans of up to several decades and that seemingly defy the commonly observed trade-off between fecundity and longevity [21–24]. Social insects (termites and ants as well as some bees and wasps) are further characterized by a reproductive division of labour: within a colony, the typically long-lived queens (and in termites, also kings) are the only reproducing individuals, while the other colony members (workers and sometimes soldiers) perform all non-reproductive tasks, such as foraging, brood care and defence, and are comparatively short-lived. Thus, as is the case in long-lived social mole-rats [25,26], reproductive individuals with exceptionally long lifespans (queens) have evolved in social insects. The convergent evolution of sociality and reproductive division of labour (‘castes’, comprising reproductives, workers and sometimes soldiers) appear to be associated with selection for long lifespans in reproductives (see also [21,24,27]). This calls for investigation of the convergent, or possibly parallel, evolution of the mechanisms underlying a long lifespan in reproductives.

Social animals are especially suited for ageing studies because both short- and long-lived phenotypes are encoded by the same genome within a colony (e.g. [17,24,28] and references therein). Indeed, outside social insects and mole-rats, such extreme (and in this case phenotypically plastic) differences in lifespan are only found in a few, distantly related taxa (e.g. [4]), which makes controlled comparisons difficult. The shared genetic background among castes within a colony furthermore means that caste-associated differences in longevity are generally not the result of genetic variation among individuals but are due to differences in gene expression. Transcriptomic studies of social insects therefore hold great promise for uncovering the physiological mechanisms underlying large differences in lifespan (e.g. [22,28,29]). To date, however, most such studies have focused on single species and not leveraged the potential power of comparative transcriptomics across taxa.

Here, we have examined the mechanisms underlying ageing in social insects by comparing gene expression patterns between young and old queens (and for termites, also kings) and workers across different social insect lineages: two termite (Blattodea, Isoptera), two bee (Hymenoptera, Apoidea) and two ant species (Hymenoptera, Formicidae) (for species and lifespan characteristics, see table 1). We studied patterns of life history and ageing of these species comparatively within a collaborative framework, the ‘So-Long’ consortium (www.so-long.org). This consortium tackles major questions about the apparent ‘reversal’ of the fecundity–longevity trade-off in the context of insect sociality by using species of different social complexity for each lineage and applying standardized methods when technically feasible. However, major biological differences among the species studied by our consortium sometimes necessitated the use of, for example, different tissues for transcriptomic analysis since the amount and quality of tissue that could be obtained constrained our use of specific tissues. In brief, we employed gene expression data derived from transcriptomes of target species to identify putative differences and commonalities in ageing-related expression patterns across three social insect lineages, with a special focus on the TI–J–LiFe network (figure 1; electronic supplementary material, §S1.0 and table S1). By comparing our results with published work from the well-established ageing model *D. melanogaster*, we begin to uncover how long-lived social insects might differ in their molecular underpinning of ageing and life-history traits when compared with short-lived solitary insects.

**Table 1.** Overview of samples included in this study.

species	social complexity	samples (no.)	tissue used and age	mapped against
*Cryptotermes secundus* (Hill, 1925) (Isoptera)	low: ‘cooperative breeders' colony size: 200–400 individuals; totipotent workers; lifespan: queens: up to 13 years workers: at least 4 years	old (2) and young (2) queens old (2) and young (2) kings old (2) and young (2) workers (*N* = 12)	whole body (without gut) old kings and queens: >7 years young kings and queens: 1 year workers: young: <6 months old: >3 years	*Cryptotermes secundus* (draft) genome
*Macrotermes bellicosus* (Smeathman, 1781) (Isoptera)	high: ‘superorganisms’ colony size: a few million individuals; two sterile worker castes; lifespan: queens: up to 20 years workers: 2–3 months	old (3) and young (4) queens old (3) and young (2) kings old (3) and young (3) minor workers old (3) and young (3) major workers (*N* = 24)	head + prothorax^a^ kings queens: young: <3 years old: >9 years young: 1 year workers: young: <1 month old: 2–3 months	*Macrotermes natalensis* v. 1.0
*Euglossa viridissima* (Friese, 1899) (Apiodea, Hymenoptera)	low: ‘facultative eusocial’ colony size: 1–5 individuals; totipotent workers; lifespan: queens: 2–6 months workers: 2–6 weeks	old (6) and young (4) queens old (3) and young (4) workers (*N* = 17)	abdomen queens: young: <1.5 months old: >1.5 months workers: young: <3 weeks old: >3 weeks	*Euglossa dilemma* v. 1.0/*Apis mellifera* v. 4.5
*Apis mellifera capensis* (Escholtz, 1821) (Apoidea, Hymenoptera)	high: ‘superorganism’ colony size: 10 000–60 000 individuals; functionally sterile workers; lifespan: queens: 1–5 years workers: 4–6 weeks	pseudo-queens, early stage (2) pseudo-queens, late stage (2) workers, early stage (2) workers, late stage (2) (*N* = 8)	fat body pseudo-queens: young: 3 and 4 days old: 7 and 8 days workers: young: 3 and 4 days old: 7 and 8 days	*Apish mellifera* v. 4.5
*Platythyrea punctata* (Smith, 1858) (Formicidae, Ponerinae, Hymenoptera)	low: ‘clonal ant’ colony size: 30–80 individuals; totipotent workers; lifespan: reproductive worker: >400–500 days non-reproducing workers: around 200 days	old (5) and young (5) dominants: head old (5) and young dominants (5): abdomen^b^ old (5) and young subordinates (5): head old (5) and young (5) subordinates: abdomen^b^ (*N* = 40)	head, gaster dominants: young: 17 days old: 112 days subordinates: young: 17 days old: 112 days	*de novo* assembly
*Temnothorax rugatulus* (Emery, 1895) (Formicidae, Myrmicinae, Hymenoptera)	high: ‘superorganisms’ colony size: 50–500 individuals; functionally sterile workers; lifespan: queens: probably 15–20 years workers: probably 2–3 years	old (8) and young (8) queens: brain old (8) and young (8) queens: fat body^b^ old (6) and young (6) workers: brain old (6) and young (6) workers: fat body^b^ (*N* = 56)	brain, fat body queens: young: <3 months, old: approx. >5 years workers: young (nurse): <1 year old (forager): >1 year; estimated by task	*de novo* assembly

^a^In [30], the same samples were referred to as ‘head’, yet the prothorax was attached to the head.

^b^Since the results were comparable to those for head (*P. punctata*) and fat body (*T. rugatulus*), PCA plots for the gaster of *P. punctata* and for the brain of *T. rugatulus* are provided in electronic supplementary material, figures S13 and S16; annotations are provided in electronic supplementary material, tables S10 and S13.

## 2. Material and methods

### (a) Choice of study species

For comparative gene expression analyses, we selected two termites, two bees and two ants (table 1). For each lineage, we studied one species with low and one species with high social complexity. Low social complexity implies small colonies, limited caste differences and workers that can become reproductives, while highly complex colonies are typically large, have marked caste differentiation and workers that cannot fully replace a queen (i.e. are functionally sterile). Species selection was influenced by accessibility and prior knowledge on the biology of the species.

Among the termites, we chose the wood-dwelling termite *Cryptotermes secundus* (Kalotermitidae), a species with low social complexity. It nests in dead trees, which serve as food and shelter, and workers never leave the nest to forage [31]. Kings and queens develop from workers; the latter are totipotent immatures [31–33]. This species was contrasted with the fungus-growing termite *Macrotermes bellicosus* (Termitidae, Macrotermitinae). *Macrotermes bellicosus* has long-lived, highly fecund queens and kings and two sterile worker castes: major and minor workers [30].

From among the bees (Apoidea), we chose *Euglossa viridissima* as a representative of low social complexity. It is a facultatively eusocial species in which all nests are initiated by a solitary foundress female. Once the first brood has emerged, one or several females from this first brood can remain in the nest and help the foundress female to raise a second brood, thus initiating the shift to a social nest structure in which the foundress/mother is dominant and one or more daughters are subordinate [34,35]. Females of this species are totipotent; a subordinate female can take over the role of dominant upon the mother's death or departure from the nest [36]. As a second bee species we chose the obligate eusocial honeybee *Apis mellifera capensis*. A honeybee colony usually consists of several thousand sterile workers and a single, highly fecund queen. Workers of this subspecies are characterized by the peculiarity that they can develop into highly fecund pseudo-queens that produce genetically near-identical offspring by automixis [37,38].

For the ants, we used the clonal ant *Platythyrea punctata* as representative of a species with low social complexity. Colonies are small, and workers can clonally produce identical female offspring from unfertilized eggs by thelytokous parthenogenesis [39]. Despite the absence of morphological or genetic differences among totipotent nest-mates, colonies are characterized by a reproductive division of labour between one (occasionally several) reproductively dominant workers (hereafter, dominants) and a majority of subordinate, non-reproducing workers (hereafter, subordinates) [40]. As an ant with high social complexity, we studied *Temnothorax rugatulus*, which nests in rock crevices or acorns. *Temnothorax rugatulus* colonies have a standard caste diphenism, and colonies consist of around 150–250 functionally sterile workers and one or several queens [41–44]. Additional information is provided in electronic supplementary material, §S1.1.

### (b) Data sampling

We collected gene expression data from transcriptomes of young and old individuals of reproducing castes (queens, pseudo-queens, reproducing workers and, for termites, also kings) as well as non-reproducing workers from recently published studies of our So-Long consortium for two termites (*C. secundus* and *M. bellicosus*), two bees (*E. viridissima* and *A. mellifera capensis*) and the ant *T. rugatulus* (see [30,42,45,46], and [36] and details therein). The use of these published data partly limited our sample size. Yet, we re-analysed all data consistently across taxa, thus generating for the first time a comprehensive dataset that covers disparate social insect lineages and allows direct comparisons across species using a common, standardized analytical framework (see also electronic supplementary material, §S1.1).

Because data were only available for young and old queens of *T. rugatulus*, we complemented them with newly sequenced transcriptomes of young and old workers*.* Although transcriptome data from young individuals of *P. punctata* have been published [40], we used newly sequenced transcriptomes of this species, representing clonally identical young and old individuals from two social groups (hereafter called castes, for simplicity), i.e. dominant reproductives and subordinate non-reproductives.

In general, it is very difficult to determine the age of social insects, and this is a common problem in studies addressing social insect ageing. In our samples, young individuals were generally much younger than old individuals, although (i) the absolute lifespan differences and (ii) the accuracy of age determination differed between species (table 1). The observation that absolute lifespan differences between young and old samples varied among species in our study is consistent with the differences in maximum longevity across the species (table 1).

An overview of the samples, species and tissues used for RNA extraction and additional information such as lifespan are given in table 1, and detailed sample information is provided in electronic supplementary material, §S1.1.

### (c) Generation and processing of newly sequenced transcriptome data

To obtain new transcriptome data for *P. punctata*, we generated a total of 40 transcriptomes, comprising five from each of the following sample types: individual heads of young and old dominants; heads of young and old subordinates; and abdomens (i.e. gasters including the petiole) from these same individuals (young and old of both dominants and subordinates; although they are not true morphological castes, we refer to them as ‘castes’ in the following) (electronic supplementary material, table S2).

A total of 24 samples of young and old workers of *T. rugatulus* were used for transcriptome sequencing (brains and fat bodies separately, each from six young and six old workers; note brains and fat bodies could not be generated from the same individuals; electronic supplementary material, table S3).

Except for the two ants, for which we generated new transcriptomes, we only used a single tissue for our comparative analyses. This was mainly because of technical constraints since it was impossible to a obtain sufficient amount of high-quality RNA from other tissues.

We generated transcriptome data with paired-end sequencing using Illumina HiSeq 4000 at the Beijing Genomics Institute (BGI)-Shenzhen with a read length of 100 bp and approximately 4 Gbases of raw data per sample. Detailed information on dis§and sample processing, RNA extraction, library preparation and sequencing are described in electronic supplementary material, §S1.1.1.

Raw sequence reads were cleaned, trimmed and quality-checked (details are given in electronic supplementary material, §S1.1.2). We generated *de novo* transcriptome assemblies since genomes are not available for either ant species. We only used paired-end reads for the *de novo* assemblies. For *P. punctata*, we used all newly generated transcriptomes. For *T. rugatulus*, we used the newly generated raw sequence data from the worker samples plus the published raw sequence data from old and young queens [42] (NCBI accession no. GSE111415). *De novo* assemblies were generated with Trinity v. 2.8.4 [47] (see electronic supplementary material, §S1.1.3 for details). These *de novo* assemblies (electronic supplementary material and archive S1 in the Dryad Digital Repository [48]) were subsequently used to map raw sequence reads and to obtain read count estimates for all the samples of both species. All newly sequenced raw sequence reads have been deposited at the NCBI Sequence Read Archive (SRA) under BioProject Accessions PRJNA672962 (*P. punctata*) and PRJNA682352 (*T. rugatulus*); see also electronic supplementary material, tables S2 and S3.

### (d) Estimation of read counts for new and published data

For both ants, we used the RSEM approach v. 2.15 [49], as implemented in Trinity v. 2.8.4, to obtain read count estimates for each contig and sample and then introduced the read counts into a single estimated count matrix for each of the species (for details see electronic supplementary material, §S1.1.3). Note that for both ant species we used the transcript level because an assignment to gene level is unreliable when only based on the Trinity gene prediction and when gene models are not available. In the following, we therefore use ‘gene/transcript’ when referring to termites and bees (genes) versus ants (transcripts), respectively. Read count matrices (electronic supplementary material; archive S2 in Dryad [48]) served as input for principal component analyses (PCAs) performed in R v. 3.4.4 [50], as implemented in DESeq2 [51]. Read count tables for the two termites, *C. secundus* and *M. bellicosus*, and the two bees, *E. viridissima* and *A. mellifera capensis*, were extracted from published studies and/or received from the authors directly [30,36,45,46]. For *C. secundus* and *E. viridissima*, the original read count tables at the transcript level were converted into the gene level with the R packages ‘tximport’ and ‘tximportData’ [52], using gene model information from the published genomes of *C. secundus* [53] or *Euglossa dilemma* [36,54]. Detailed information is provided in electronic supplementary material, §S1.1.4.

### (e) Comparing gene expression using principal component analyses

To perform quantitative comparisons across species, we chose to perform PCAs. This approach allowed us to identify genes/transcripts separating biological groups (caste and age) for each species. This method is especially suited for our dataset, for which differences in sample sizes among species would have confounded differential gene expression analyses because sample size is critical for identifying significantly differentially expressed genes (see [55]).

To compare gene/transcript expression profiles across taxa, we performed PCAs separately for each species and tissue (for the ants, we additionally analysed both tissues combined) by using curated, variance-stabilized, species-specific read count tables derived from the transcriptome data. We generated PCA plots for the first and the second principal components (PC1, PC2) with the R package ‘matrixStats’ v. 0.56 [56], considering the top 500 genes/transcripts contributing to the PCA. PCA plots revealed whether samples were grouped by caste and/or age along the PCs. To test for significant effects of caste, age and their interaction on the scores of PC1 and PC2, we used type II analysis of variance (ANOVA) models (separately for each species; and for the ants, also separately for each tissue as well as for both tissues combined; for the latter, we added tissue as an additional factor) in R v. 3.6.3. For each species, we extracted the 100 genes/transcripts with the highest loadings (i.e. the 50 genes/transcripts with the most positive and 50 with the most negative loadings; hereafter top ±50 genes/transcripts) on PC1 and PC2. This allowed us to associate genes/transcripts with castes and/or age and compare patterns across species (electronic supplementary material, §S1.2). We also extracted additional PCs beyond PC1 and PC2 and analysed their potential associations with age and caste (electronic supplementary material, §S1.2). However, they were less informative as they captured much less of the variance in the data (see electronic supplementary material, §S1.2, figure S1 and table S4). In the following, we thus concentrate on PC1 and PC2.

### (f) Functional annotation and gene ontology enrichment of the top ±50 genes/transcripts from principal components 1 and 2

We obtained functional annotations for the top ±50 genes/transcripts contributing to PC1 and PC2 identified in the PCA from the genome annotations that had been used to estimate the read counts, i.e. annotations from *C. secundus* (see [45]), *Macrotermes natalensis* (see [30]), *E. dilemma* (see [36,54]) and *A. mellifera* (see [46]). To do so, we used BLAST searches [57] implemented in the BLAST suite v. 2.9+ ([58]; see settings below and in electronic supplementary material, §S1.3). We used amino acid sequences from the respective genomes (termites and bees) and transcript sequences at the nucleotide level (ants) from the respective *de novo* assemblies as a query to search against the non-redundant NCBI database. The search was restricted for both termites to the termite *Zootermopsis nevadensis*, which has the best annotated termite genome*.* For *E. viridissima*, we searched against *A. mellifera.* Sequences from *P. punctata* and *T. rugatulus* were searched against the ants *Harpegnathos saltator* and *Solenopsis invicta*, respectively, i.e. the most closely related species with fully available genomes and well-curated annotations. The threshold *e*-value was set to 10^−5^, considering only the best hit with the smallest *e*-value and the best bit score along with its annotations. In addition, for all species we performed BLAST searches against *D. melanogaster* (release 6.27) using the same settings as above and extracted annotations for the best matching hits from FlyBase ([59]; release FB_2020_03, June 2020; http://flybase.org). Finally, we ran InterProScan [60] v. 5.4.4–79.0 on all top ±50 sequences at the protein level (termites and bees) or at the nucleotide level (ants) using default settings. Further details are given in electronic supplementary material, supplementary text and tables S5–S14. Additionally, we performed gene ontology (GO) enrichment analyses to classify the top ±50 genes/transcripts of PC1 and PC2 (electronic supplementary material, §S1.4, and archives S3 and S4 in Dryad [48]). We did not find a consistent pattern across species, except for the termites (electronic supplementary material, §1.4 and tables S15–20). As a caveat, it should be noted, however, that genes/transcripts associated with the TI–J–LiFe network, especially those associated with JH biosynthesis and signalling, are generally not well annotated with respect to GO terms. This might render strict tests of GO enrichment of TI–J–LiFe network genes difficult.

### (g) Differential expression analyses and overlap with principal component analysis results

To further probe the robustness of our results, we performed differential gene/transcript expression analyses using DESeq2 [51] and examined the effects of caste, age and their interaction on gene/transcript expression (electronic supplementary material, §S1.5, and archive S5 in Dryad [48]). We then examined whether the overlap between the top ±50 genes/transcripts (PC1 and PC2) that showed effects of age or of age-by-caste interaction in the PCA overlapped with the top 100 most strongly differentially expressed genes/transcripts (according to the adjusted *p*-values) between young and old individuals as identified in the DESeq2 analyses (electronic supplementary material, §§S1.5 and S1.6, and archive S5 in Dryad [48]).

### (h) Identification of TOR/IIS–JH–LiFe genes among the top genes/transcripts

Annotations of the top ±50 genes/transcripts were explored manually using publicly available information from the literature and from FlyBase to identify shared genes. Additionally, genes/transcripts were carefully checked by hand curation for the following functional categories: fecundity-related (e.g. vitellogenins), JH biosynthesis, immunity, chemical communication and transposable elements (TEs). To do so, all annotation information retrieved from BLAST and InterProScan was used to identify genes involved in these functions (e.g. genes from immune pathways, or genes known to be involved in JH biosynthesis or JH signalling) and associated with the corresponding function. Particular attention was paid to the 123 genes known from *D. melanogaster* belonging to the TI (TOR/IIS) and J (JH biosynthesis and signalling) components of the TI–J–LiFe network (electronic supplementary material, table S1; also see figure 1). To perform a systematic search, we checked whether these 123 genes were included in the top ±50 sequences (electronic supplementary material, tables S5–S14) based on their assigned FlyBase gene IDs. The TI–J–LiFe genes for which we found homologues among the top ±50 sequences are given in electronic supplementary material, tables S5–S14, last column.

We then tested whether TI–J–LiFe homologues were enriched among the top ±50 genes/transcripts. This was only possible for the 123 TOR-, IIS- and JH-related genes (electronic supplementary material, table S1) as no concrete gene lists exist for downstream components. We first determined how many homologues of these 123 candidate genes from *D. melanogaster* occurred in each study species by performing BLAST searches using the corresponding *D. melanogaster* genes as query either against the official gene sets from available genomes that had been used to map the raw sequence reads (i.e. termites and bees) or against the *de novo* assemblies (for the ants) (see electronic supplementary material, §S1.7 and table S21, and archive S6 in Dryad [48]). Next, we used contingency analyses to test whether the number of TI–J–LiFe homologues identified among the top ±50 genes/transcripts (indicated in electronic supplementary material, tables S5–S14, last column) was significantly higher than expected based on the number of TI–J–LiFe homologues occurring in each species (electronic supplementary material, table S21). We also calculated the probability that the observed overlap of TI–J–LiFe homologues occurred by chance using a hypergeometric distribution.

### (i) Supplementary analyses

#### (i) Identification of single-copy orthologues

An alternative approach for our analyses would have been to concentrate on orthologues instead of all genes/transcripts. To evaluate the potential usefulness of such an approach, we determined shared single-copy orthologues (SCOs) among all study species and *D. melanogaster* and then identified them among our top ±50 PC genes/transcripts (details of orthology inference are given in electronic supplementary material, §S1.8; see also electronic supplementary material, and archive S7 in Dryad [48]).

These analyses revealed only few SCOs. Depending on the species, only between 3.6 and 7.4% of the genes/transcripts were SCOs (see archive S7 in Dryad [48]). This is only a minor proportion of all genes/transcripts. We therefore decided to base our PCA analyses on all genes/transcripts since using only SCOs would imply a considerable loss of information.

Similarly, among our top ±50 genes/transcripts (PC1 and PC2), there were only few SCOs (see Results and electronic supplementary material, tables S5–S14). Depending on the species and PC, only between 1 and 14 of the top ±50 PC genes/transcripts were SCOs (electronic supplementary material, tables S5–S14, and for an overview see electronic supplementary material, table S22).

#### (ii) Vitellogenin gene trees

Our results pointed to the importance of *vitellogenins* (*Vg*s) or *Vg-like* genes. As these genes have been found in social hymenopterans to play additional roles that are unrelated to reproduction [61–66], we did a gene tree analysis with the aim of obtaining more information on the potential function of these genes. We inferred a maximum-likelihood gene tree that included all identified *Vg*s or *Vg-like* sequences from our six study species as well as data from the termite *Z. nevadensis*, the cockroach *Blattella germanica* and a subset of species used in Kohlmeier *et al.* [63] (see electronic supplementary material, tables S23 and S24; details are described in the electronic supplementary material, §S1.9 and in archive S8 in Dryad [48]).

## 3. Results and discussion

**Figure 2.**
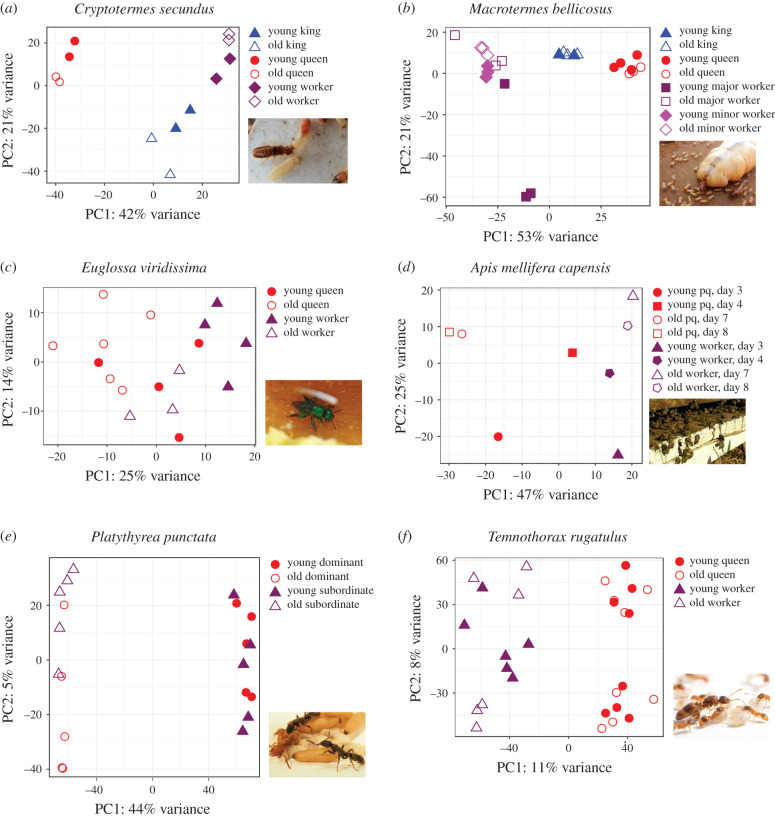
Results of PCAs of variance-stabilized RNA read counts of young and old individuals. Shown are the first (*x*-axis) and second (*y*-axis) PCs with variance explained by each PC. Each point represents the expression profile of one individual/sample for 500 genes/transcripts. Filled shapes represent young and unfilled shapes old individuals: (*a*) the termite *C. secundus* (photo: J. Korb), (*b*) the termite *M. bellicosus* (photo: J. Korb), (*c*) the bee *E. viridissima* (photo: A. C. Séguret), (*d*) the bee *A. mellifera capensis*, pq: pseudo-queens (photo: V. Dietemann), (*e*) the ant *P. punctata* (head samples) (photo: A. Bernadou) and (*f*) the ant *T. rugatulus* (fat body samples) (photo: R. Libbrecht). (Online version in colour.)

Differences in gene/transcript expression between castes of social insects (e.g. workers versus reproductives) are expected to capture substantial information, or ‘signal’, not only because of their different forms and functions within the colony but also because of their markedly different lifespans and somatic maintenance patterns. Additionally, ageing-related physiological processes are captured by contrasting young versus old individuals within a caste. While we cannot rule out the possibility of false negatives, we found strong effects of caste on gene expression in all six species (for PC1 and PC2), whereas age affected expression only in four species (figure 2 and table 2; electronic supplementary material, tables S5–S10, S12 and S13). The absence of an age signal in *T. rugatulus* and the weak trend in *A. mellifera capensis* might be explained by the fact that age could not be very reliably determined in the former and that age differences and sample sizes were small in the latter (table 1). Interestingly, the effects of age and caste interacted with each other in the four species with an age effect: *C. secundus*, *M. bellicosus*, *E. viridissima* and *P. punctata*. This suggests that some genes that are downregulated during the life of a reproductive are upregulated when workers become older (or *vice versa*). Before discussing shared, general patterns across species, we first present species-specific results.

**Table 2.** Results of ANOVA tests of the effects of age, caste and their interaction on PC1 and PC2. Shown are the degrees of freedom (d.f.), *F*- and *p*-values from type II ANOVAs testing the effects of age, caste and the interaction between age and caste for each species, separately for PC1 and PC2. Note that, for the ants, two tissue sources were studied.

	age			caste			age × caste		
	d.f.	*F*	*p*	d.f.	*F*	*p*	d.f.	*F*	*p*
*C. secundus*	*whole body without gut*								
PC1	1	5.09	0.065	2	432.21	<0.0001	2	3.30	0.108
PC2	1	2.22	0.187	2	43.26	0.0003	2	7.17	0.026
*M. bellicosus*	*head + prothorax*								
PC1	1	3.17	0.094	3	191.92	<0.0001	3	4.21	0.022
PC2	1	10.79	0.0047	3	5.38	0.009	3	6.73	0.004
*E. viridissima*	*abdomen*								
PC1	1	13.05	0.003	1	13.99	0.002	1	0.14	0.718
PC2	1	0.02	0.900	1	0.09	0.771	1	7.35	0.018
*A. mellifera capensis*	*fat body*								
PC1	1	2.80	0.170	1	44.73	0.003	1	6.45	0.064
PC2	1	7.65	0.051	1	0.003	0.961	1	0.50	0.520
*P. punctata*	*head*								
PC1	1	5945.57	<0.0001	1	0.04	0.836	1	1.72	0.209
PC2	1	0.001	0.977	1	2.92	0.107	1	6.39	0.022
	*abdomen*								
PC1	1	526.58	<0.0001	1	28.87	<0.0001	1	15.39	0.001
PC2	1	7.72	0.013	1	40.17	<0.0001	1	13.90	0.002
*T. rugatulus*	*brain*								
PC1	1	0.74	0.397	1	972.39	<0.0001	1	10.84	0.370
PC2	1	0.01	0.915	1	0.03	0.854	1	0.002	0.960
	*fat body*								
PC1	1	0.34	0.566	1	309.61	<0.0001	1	0.17	0.678
PC2	1	0.02	0.896	1	0.089	0.768	1	<0.001	0.997

### (a) Taxon-specific patterns of gene expression

#### (i) Termites

The transcriptomes of whole animals (minus guts) of the wood-dwelling termite *C. secundus* revealed that PC1 was related to caste and explained 42% of the variance. Along PC1, queens had significantly lower values than kings, which in turn had significantly lower values than workers (table 2; electronic supplementary material, table S5 and figure S2). PC2 explained 21% of the variance and separated kings, which had the lowest values, from the other castes; moreover, it revealed a significant interaction between age and castes (table 2 and figure 2*a*). Young queens and kings had higher values than older ones, whereas this pattern was reversed for workers (figure 2*a*; electronic supplementary material, figure S3). The top −50 genes with the strongest negative loadings on PC1 (characterizing queens, and partly kings) included three conventional *Vg*s, six histone-related genes and several genes related to E3 ubiquitin proteins (electronic supplementary material, table S5). The latter finding is interesting since, in the nematode worm *Caenorhabditis elegans* and in *D. melanogaster*, E3 ubiquitin ligases have been found to regulate lifespan [67,68]. By contrast, the top +50 genes with the strongest positive loadings (characterizing workers) comprised two TEs, three genes potentially linked to chemical communication and a *JH epoxidase* that is likely to be worker-specific [69]. The top −50 genes with the strongest negative loadings on the age/caste-related PC2 (characterizing kings, old reproductives and young workers) included two antimicrobial peptide genes (AMPs) involved in immunity, one *transferrin* potentially representing an antioxidant (see [70]) and one gene probably linked to chemical communication. The top +50 genes with positive loadings on PC2 (characterizing old workers and young reproductives) included one TE and one immune-related gene, the gene *gram-negative bacteria-binding protein, GNBP*.

For the head plus prothorax transcriptomes of the fungus-growing termite *M. bellicosus*, PC1 mainly separated castes and explained 53% of the variance, whereas PC2 mainly separated the age groups, especially in major workers, explaining 21% of the variance (figure 2*b*). Along PC1, major and minor workers had significantly lower values than kings, which had significantly lower values than queens (table 2) (electronic supplementary material, figure S4). Along PC2, samples were significantly separated by age (table 2). While this effect was not apparent in reproductives, young workers had significantly lower values than old workers, especially in major workers (table 2 and figure 2*b*; electronic supplementary material, figure S5). Genes with large negative loadings on PC1 (characterizing workers) included at least four genes related to chemical communication (electronic supplementary material, table S6). Genes with large positive loadings on PC1 (characterizing reproductives) included three conventional *Vg*s. We also observed a signature of high JH biosynthesis, indicated by the expression of a queen-specific *JH epoxidase* gene and two *hydroxymethylglutaryl-co-enzyme* (*HMG CoA*) *synthase* genes, all with large positive loadings on PC1. One *Vg* and two TEs had large positive loadings on PC2 and were associated with old age, especially in major workers. By contrast, two genes related to chemical communication were positively associated with young age.

#### (ii) Bees

For the abdominal transcriptomes of the facultatively eusocial bee *E. viridissima*, we found that PC1 was associated with both caste and age and explained 25% of the variance (figure 2*c*). PC2, explaining 14% of the variance, revealed an interaction between caste and age. Along PC1, old individuals had significantly lower values than young individuals and queens had significantly lower values than workers (table 2; electronic supplementary material, figures S5–S7). Along PC2, there was a significant interaction between caste and age, with young queens having lower values than old queens, whereas this pattern was reversed in workers (table 2; electronic supplementary material, figure S8). Genes associated with queens and old age (i.e. with large negative loadings on PC1) included genes annotated as *major royal jelly protein* (*mrjp*), several immune-related genes (e.g. four AMP genes, *chymotrypsin*), a *transferrin* (also see above) and several histone-related genes (electronic supplementary material, table S7). Genes with the largest positive loadings on PC1 did not show a striking pattern. Genes characterizing young queens and old workers (i.e. large negative loadings on PC2) included one *GNBP*, a *vitellogenin-like gene* (*Vg-like A*), a phenoloxidase-related gene and *transferrin*. Among the genes associated with old queens and young workers (i.e. large positive loadings on PC2) were genes annotated as *mrjp* and *slimfast* from the TOR pathway.

Analysis of the fat body transcriptomes of the honeybee *A. mellifera capensis* revealed that PC1 explained 47% of the variance and separated the ‘castes’, with pseudo-queens having significantly lower values than workers (table 2 and figure 2*d*; electronic supplementary material, figure S9). PC2 differentiated the age groups, with old individuals having significantly higher values than young ones (table 2; electronic supplementary material, figure S10), and explained 25% of the variance (figure 2*d*). Genes associated with workers, i.e. with large positive loadings on PC1, included reproduction-related genes (*Vg*, *mrjp*), two genes involved in JH biosynthesis (*farnesol dehydrogenase*, *juvenile hormone acid methyltransferase*) and at least five genes associated with chemical communication, including cuticular hydrocarbon (CHC) production as well as perception (electronic supplementary material, table S8). The association of *Vg* and *mrjp* with workers appears surprising but might be explained by the fact that worker honeybees produce high amounts of royal jelly as brood food. This is consistent with the notion that *Vg* has become disassociated from reproduction in the honeybee [71,72] and could also serve as an immune effector [66]. Genes associated with pseudo-queens (i.e. with large negative loadings on PC1) revealed a clear immune signature (including three AMP genes, *GNBP1*, and two serpin-related genes) as well as two olfactory receptor genes. Among the genes positively associated with old age (large positive loadings on PC2), we found several genes related to immunity (one AMP, one *GNBP1-2* precursor and *peptidoglycan recognition protein 1*), reproductive physiology (an *mrjp* precursor), JH biosynthesis (*juvenile hormone acid methyltransferase*) and three genes involved in chemical communication. Genes associating with a young age (large negative loading on PC2) also included six genes related to chemical communication.

#### (iii) Ants

For head transcriptomes of the clonal ant *P. punctata*, PC1 clearly separated young from old individuals, with young individuals showing significantly higher values than old ones (table 2; electronic supplementary material, figure S11); it explained 44% of the variance (figure 2*e*). PC2 only explained 5% of the variance and revealed an interaction between caste and age (electronic supplementary material, figure S12). Old dominant individuals had significantly lower values than young dominants, while the reverse was true for subordinates (table 2). Genes related to old age (strong negative loadings on PC1) included three conventional *Vg* transcripts (electronic supplementary material, table S9), and these genes were also associated with old dominant individuals along PC2 (large negative loadings on PC2). Similarly, one gene of the TOR pathway (annotated as encoding TOR complex 2 subunit MAPKAP1 isoform X2) was associated with old dominant individuals (electronic supplementary material, table S9). Additional data for gaster transcriptomes can be found in electronic supplementary material, figure S13 and table S10. They were more difficult to interpret as both PCs were associated with age, caste and an interaction between age and caste (table 2).

The combined analysis of both tissues (electronic supplementary material, table S11) revealed that both PC1 and PC2 were significantly associated with age and tissue (table 3 and electronic supplementary material, figure S14a). Thus, we also retrieved a clear age signal when combining both tissues.

**Table 3.** Results of ANOVA tests of both ants and of the effects of age, caste, tissue and their interaction on PC1 and PC2 for the two ant species. Shown are the percentage of variation (var.) explained by the respective PC and type II ANOVA results (degrees of freedom: d.f.; *F*- and *p*-values) testing the effects of age, caste, tissue and their interaction for each ant species, separately for PC1 and PC2.

	*P. punctata*		*T. rugatulus*	
	PC1	PC2	PC1	PC2
% var.	67	18	50	8
age				
d.f.	1	1	1	1
*F*	11.3	14 112	0.1	0.8
*p*	0.002	<0.001	0.74	0.37
caste				
d.f.	1	1	1	1
*F*	0.2	0.3	1.7	459
*p*	0.64	0.58	0.20	<0.001
tissue				
d.f.	1	1	1	1
*F*	15 739	11	318	2.1
*p*	<0.001	0.002	<0.001	0.15
age × caste				
d.f.	1	1	1	1
*F*	0.02	2	0.1	0.1
*p*	0.90	0.17	0.75	0.82
age × tissue				
d.f.	1	1	1	1
*F*	8	14	2.5	1.9
*p*	0.008	<0.001	0.12	0.17
caste × tissue				
d.f.	1	1	1	1
*F*	0.8	0.1	7.5	155
*p*	0.37	0.80	0.008	<0.001
age × caste × tissue				
d.f.	1	1	1	1
*F*	0.1	1	0.7	1.9
*p*	0.74	0.29	0.41	0.17

Analysis of the fat body transcriptomes of the socially complex ant *T. rugatulus* showed that PC1 clearly separated castes, with queens showing significantly higher values than workers (table 2 and figure 2*f*; electronic supplementary material, figure S15); it explained 11% of the variance. PC2 only explained 8% of the variance and could not be clearly associated with age or caste (table 2). This finding is similar to a study of the closely related species *Temnothorax longispinosus*, for which caste also played a stronger role than age in explaining gene expression patterns [73]. Genes characterizing queens (large positive loading on PC1) included two transcripts annotated as conventional *Vg*s, two annotated as myrmicine *Vg*s and four involved in chemical communication (electronic supplementary material, table S12). Genes associated with workers (strong negative loading on PC1) included a transcript from the JH biosynthesis pathway, annotated as *farnesyl pyrophosphate synthase* (*FPPS*). Brain transcriptomes showed a similar pattern to those from fat bodies; PC1 was associated with caste, while PC2 could again not be associated with age or caste (electronic supplementary material, figure S15 and table S13).

When combining both tissues for the analysis (electronic supplementary material, table S14), we found that PC1 was significantly associated with tissue, whereas PC2 clearly separated the samples by caste (table 3; electronic supplementary material, figure S14b). Thus, PC2 revealed the same caste signal as for the tissues analysed separately.

We also tested whether the top ±50 genes/transcripts (PC1 and PC2) that showed effects of age or of an age-by-caste interaction in the PCA overlapped with the top 100 genes/transcripts that were most strongly differentially expressed (according to the adjusted *p*-values) between young and old individuals, as identified in our DESeq2 analyses (electronic supplementary material, §S1.6). The number of overlapping genes/transcripts was small, but we found a significantly larger overlap than expected by chance. This confirms that our results are robust (for details see electronic supplementary material, §S2.1 and table S25).

### (b) Shared transcriptomic signatures of caste and age among social insect taxa

We identified many ‘macroscopic’ commonalities across taxa at the functional and pathway levels, for example, genes involved in reproductive physiology, JH signalling and somatic maintenance functions such as innate immunity and oxidative stress. Several of them seem to be associated with the TI–J–LiFe network (figure 1).

Consistent with our hypothesis that the TI–J–LiFe network plays a major role in social insect ageing and life history, we found support for a significant enrichment of TI–J–LiFe genes in some species (electronic supplementary material, table S26). However, some species did not reveal a significant enrichment although we expected it (e.g. for the two bees). This seems to provide only partial support for our conclusions, yet it does not invalidate them: first, lack of a significant enrichment for genes of certain functions/processes does not mean that a given function/process is unimportant. Such analyses can only test for a numerical enrichment of homologues, but not their functional relevance: a single gene might have a large impact on physiology and organismal function, for example, when it is a major molecular switch or a systemic signal (this might be especially true for gene products involved in endocrine signalling). Second, our list of ‘core’ TI–J–LiFe genes (electronic supplementary material, table S1) does not cover the very large number of downstream components and transcriptional targets (LiFe) of this network. Our analyses of the top ±50 genes/transcripts clearly point to such downstream components playing an important role: we identified many downstream processes and target genes that are empirically known to be regulated by the upstream core components of TOR, IIS and/or JH signalling (e.g. the fecundity-related Vgs and yellow proteins).

Admittedly, we might have missed some signals owing to low sample size or tissue heterogeneity and therefore cannot exclude false negatives; however, the shared patterns we have identified suggest—conservatively—that there are some major commonalities across the species analysed.

#### (i) Marked absence of canonical IIS/TOR signalling components

A common pattern in our data was the marked absence (with a few exceptions) of core signalling components of the upstream part of the TI–J–LiFe network (figure 1), i.e. members of TOR and IIS pathways (e.g. the insulin-like peptides or ILPs, the insulin-like receptor InR, the central transcription factor FOXO or the kinase TOR; see electronic supplementary material, tables S5–S10, S12 and S13), despite the physiological importance of these pathways in regulating ageing and life history in solitary insects (e.g. [6–13]). This absence is noteworthy since major members of the IIS/TOR pathway, for example ILPs, are associated with reproduction and queen differentiation in ants [64,74–76] (for recent reviews [55,77], but see also [78]) and are downregulated in old honeybee queens when compared with old workers [79]. While the lack of a strong signature of canonical IIS/TOR genes in our data could potentially be due to false negatives because of small sample size and/or tissue heterogeneity, the pronounced absence of these major IIS/TOR signalling components is in good agreement with a recent study by Warner *et al.* [80], who compared transcriptomic profiles between the pharaoh ant, *Monomorium pharaonis*, and *A. mellifera*. They concluded that, beyond the core genes of the reproductive ground plan, most caste-associated genes seem to be highly plastic and presumably markedly species-specific in their expression. Our finding is also echoed by observations from *D. melanogaster*, for which two recent genomic studies have found only little evidence for genetic changes in the IIS/TOR pathway underpinning evolutionary changes in longevity [81,82]. Instead, one of these studies found a major contribution of immunity genes to the evolution of long life ([81]; see also [6] for discussion).

#### (ii) Juvenile hormone pathway genes with potentially pleiotropic life-history effects

In contrast to the TOR/IIS components of the TI–J–LiFe network, we found a clear signature of the JH pathway, the central part of the TI–J–LiFe network (figure 1), in our data. We identified genes of the JH pathway in several species, especially genes involved in JH biosynthesis (see electronic supplementary material, tables S5–S10, S12 and S13), such as *HMG CoA synthase*, *FPPS*, *farnesol dehydrogenase*, *juvenile hormone acid methyltransferase* and *JH epoxidase*. JH is a major pleiotropic hormone with gonadotropic, immunosuppressive, oxidative stress-inducing and lifespan-shortening effects in a variety of insects, including *D. melanogaster* [13–15,17,83,84]. There is also a rich body of literature that implicates JH in caste determination and behavioural maturation in social insects (e.g. [64,85–88] and references therein). We failed to detect a significant GO enrichment for JH genes. Yet these genes are not well annotated in terms of GO (i.e. there is no proper GO term for JH signalling).

#### (iii) Common signature of vitellogenin genes involved in reproduction and ageing

Consistent with increased JH biosynthesis was our striking finding that the reproductives/queens of all non-bee species were characterized by conventional *vitellogenin* genes (*Vg*s). *Vg*s are best known for encoding egg yolk precursor proteins and are an important component of the ‘fecundity’ part of the TI–J–LiFe network (figure 1); they are subject to complex regulatory interactions between IIS and JH signalling (e.g. [17,18,64,79,85,87,88] and references therein; see electronic supplementary material, tables S5–S10, S12 and S13). In the honeybee, for example, JH and Vg mutually repress one another [85,89]. Although *Vg*s serve as lipid storage and transport proteins and have major gonadotropic functions in many insects, in some social insects they also play a major role in (reproductive) division of labour (e.g. [61,62,73,90–96] and references therein). According to the reproductive ground plan hypothesis, *Vg*s belong to a core set of conserved reproduction-related genes that have been co-opted from solitary ancestors and which mechanistically underlie reproductive division of labour in social insects (e.g. [97]). Moreover, in honeybees, *Vg*s exhibit a critical pro-survival function by protecting against oxidative stress; this has led to the hypothesis that *Vg*s play an important role in regulating lifespan in social insects (reviewed in [18]; also see [72,79,98]). In our dataset, both termite species had only conventional *Vg*s (see electronic supplementary material, tables S23 and S24, and figure S17). They are the only *Vg*s found in cockroaches, which include termites that have a termite-specific *Vg* duplication [92]. The head/brain transcriptomes of ants had only conventional *Vg*s for *P. punctata* and a mixture of myrmicine conventional *Vg*s and one *Vg-like C* for *T. rugatulus*. Abdomen and fat body transcriptomes of ants and bees contained a mixture of conventional, myrmicine *Vg*s as well as *Vg-like A* and *B* (see electronic supplementary material, table S24 and figure S17). Our observations are consistent with the multi-functionality of *Vg*s and their major role in fecundity, in caste differentiation and, presumably, in the regulation of social insect ageing. Yet, a causal role for *Vg*s in affecting ageing and longevity in social insects still awaits experimental confirmation.

#### (iv) Royal jelly and other yellow proteins involved in caste determination, fecundity and longevity

Another common observation was the shared occurrence of *mrjp* (also called *royalactin*) in the bees and of the related *yellow* genes, as a function either of caste or of age (electronic supplementary material, tables S5–S10), except in the ant *T. rugatulus* (electronic supplementary material, tables S12 and S13). mrjps play a crucial role in caste determination in honeybees (mrjps are an essential component of the royal jelly), are expressed in a variety of social insects (but mostly with unknown function) and have arisen by duplication from *yellow* genes ([99,100] and references therein). In *D. melanogaster*, a single *yellow* gene affects pigmentation, sex-specific reproductive maturation and behaviour (e.g. [99,101,102] and references therein). It has been previously reported [103] that dietary supplementation with mrpj1 (royalactin) protein extends lifespan and promotes fecundity in *Drosophila* via effects on epidermal growth factor (Egf) signalling and possibly via S6 kinase, a major component of the IIS/TOR pathway; but this study is controversial (see also [104]). It will thus be important to gain a better understanding of the potential roles of mrjps and yellow proteins in social insect ageing and life history in future work.

#### (v) Somatic maintenance functions that might contribute to longevity

Genes involved in somatic maintenance also showed a strong signal in our data, i.e. genes involved in innate immunity (e.g. several *AMP*s), oxidative stress resistance (e.g. *superoxide dismutase*, *catalase* and GO terms associated with response to stress) and detoxification of phenolic plant compounds (laccases; see [105], and electronic supplementary material, tables S5–S10, S12 and S13). Many of these genes are known to be transcriptional targets of the TOR/IIS and JH pathway, and the somatic maintenance functions that they underpin are often correlated with longevity in insects (e.g. [13,81,83,106] and references therein). These genes might thus represent the ‘lifespan’ part of the TI–J–LiFe network (figure 1).

With regard to immunity, we detected a strong signal in both bees (associated with reproductives or old age) and the termite *C. secundus* (all castes and age classes), but not in the two ants (electronic supplementary material, tables S5–S10, S12 and S13). In terms of oxidative stress, we identified a few antioxidant genes associated with caste and/or old age. However, similar to previous studies (e.g. [28,107,108], reviewed in [109,110] and [111]), our results did not reveal a consistent pattern. For example, while we found *superoxide dismutase* to be expressed in queens of *T. rugatulus*, the enzyme catalase was expressed in young reproductives and old workers in *C. secundus* (electronic supplementary material, tables S5–S10, S12 and S13). Differences in the degree of social complexity might possibly account for these variable results across species (see [112]).

In addition, for termites but not for hymenopterans, we also found that the expression of TEs was associated with caste and age (see electronic supplementary material, tables S5–S10, S12 and S13). The importance of TEs for social evolution and ageing in termites has been highlighted in previous studies [30,45,53,113]. Indeed, recent evidence suggests that in *M. bellicosus*, long-lived reproductives exhibit constitutive upregulation of the piRNA pathway whose activity silences TEs and which might contribute to their longevity [30].

#### (vi) Other common patterns across social insects

Beside the above-mentioned patterns, which were all related to the TI–J–LiFe network, we also observed signatures of genes underpinning chemical communication, except in *A. mellifera capensis* and *P. punctata* (see electronic supplementary material, tables S5–S10, S12 and S13). These communication signals were not associated with a particular age or caste. Chemical communication is critically important in social insects since colony members inform each other about their colony membership, often via CHCs ([114] and references therein). Importantly, social insect queens also communicate their fertility to nest-mate workers, which hinders the workers from reproducing or killing the queen (e.g. [115–118]). Thus, it is expected that the pathways involved in chemical communication are linked to JH and indirectly to longevity. Indeed, several transcriptome studies on ageing in social insects have identified genes linked to chemical communication among fecundity/caste- and age-specifically expressed genes (e.g. [45,46]). We therefore propose to include genes related to chemical communication in the TI–J–LiFe network for social insects, because their expression is likely to be directly or indirectly regulated by JH if CHCs (or other odour-bearing compounds) function as fertility indicators in social insects.

## 4. Conclusion

Despite some experimental limitations, we found clear evidence that JH signalling and genes, regulating aspects of ageing and fecundity and belonging to the downstream part of the TI–J–LiFe network, represent a common pattern across the social insect species examined here. Conversely, we found surprisingly few canonical core components of the TOR and IIS pathways (representing the upstream part of the TI–J–LiFe network) to be associated with age or caste in our study (although we cannot rule out with certainty that this is due to false negatives). Indeed, there is a growing appreciation that genes beyond the classical and well-studied IIS and TOR core signalling components—particularly those belonging to the downstream part of the TI–J–LiFe network—might play a major but still largely underappreciated role in ageing and life history (e.g. [81]). We thus advocate for the broader TI–J–LiFe network as a more inclusive mechanistic framework for studying the molecular underpinnings of ageing, fecundity and life-history trade-offs in social insects and beyond.

## Data accessibility

Additional data can be found in the electronic supplementary material; archives are available from the Dryad Digital Repository: https://doi.org/10.5061/dryad.66t1g1k1b [48]. Newly generated transcriptome data for this study are available from NCBI, BioProject accessions PRJNA672962 (*P. punctata*) and PRJNA682352 (*T. rugatulus*).
